# Pilot Study of Effects of Intermittent Pneumatic Compression in the Immediate Peri-Operative Period on Hemodynamic Parameters in Patients After Laparoscopic Gynecologic Surgery

**DOI:** 10.3389/fsurg.2022.896452

**Published:** 2022-06-07

**Authors:** Yanchang Liu, Xuhong Tan, Yujin Cheng, Baojun Wang, Hanyu Zhang, Lili Zhang, Danyong Liu, Xiaofei Qi

**Affiliations:** ^1^ Operation Room, Shenzhen Maternity & Child Healthcare Hospital, The First School of Clinical Medicine, Southern Medical University, Shenzhen, China; ^2^ Department of Anesthesiology, Shenzhen Maternity & Child Healthcare Hospital, The First School of Clinical Medicine, Southern Medical University, Shenzhen, China

**Keywords:** intermittent pneumatic compression device, gynecologic surgery, laparoscopy, hemodynamics, arterial blood pressure, heart rate, systemic vascular resistance

## Abstract

The randomized controlled study investigated the impacts of immediate peri-operative Intermittent pneumatic compression (IPC) on hemodynamic indicators in patients undergoing laparoscopic gynecologic surgery. Patients scheduled for elective laparoscopic gynecologic surgery were randomized to control (IPC not used), pre-operative IPC, post-operative IPC, and peri-operative IPC (performed both before and after surgery) groups. Systolic blood pressure (SBP), mean blood pressure (MBP) cardiac output (CO), heart rate (HR) and systemic vascular resistance (SVR) were measured at different time points. The results showed that SBP changes not obviously over time in the control and peri-operative IPC group. Compared with values before surgery, the pre-operative IPC group had a lower SBP (*P *< 0.01) at the end of PACU stay, whereas the post-operative IPC group had a higher SBP (*P *< 0.01) after surgery. All groups exhibited little or no variation in HR, CO and SVR. Conclusion is peri-operative IPC has no major adverse effects on hemodynamic parameters.

## Introduction

Laparoscopic surgery is widely used in the treatment of many gynecologic diseases (1). However, venous thromboembolism (VTE), which includes deep vein thrombosis (DVT) and pulmonary embolism, remains a preventable cause of morbidity and mortality after laparoscopic surgery for gynecologic conditions. Previous studies have reported VTE rates of 0.6%–11.5% after laparoscopic gynecologic surgery (2–6). Factors associated with an increased risk of DVT after laparoscopic gynecologic surgery include older age, hypertension, higher levels of D-dimer, longer duration of surgery, higher intraoperative pneumoperitoneum pressure and longer bed rest time (3, 4, 7). Notably, more than 40% of deaths after gynecologic surgery are attributable to VTE (8), highlighting the need for preventive strategies in high-risk cases.

Various methods are available to reduce the risk of DVT in patients undergoing gynecologic surgery, including pharmacologic and mechanical prophylaxis (8). The pharmacologic strategies include unfractionated heparin, low molecular weight heparin (LMWH; e.g., enoxaparin), thrombin inhibitors (e.g., argatroban) and factor Xa inhibitors (e.g., apixaban) (9–11). However, these agents are associated with potentially serious adverse effects such as postoperative hemorrhage (for all agents), heparin-induced thrombocytopenia and apixaban-induced liver injury (8). The mechanical methods for preventing VTE include graduated compression stockings and intermittent pneumatic compression (IPC) devices (12). Although graduated compression stockings are widely employed to reduce the risk of DVT after gynecologic surgery, their use alone is not recommended for patients at high risk of VTE (13). Furthermore, the Asian VTE guidelines do not recommend the use of graduated compression stockings (14).

IPC is performed using devices that apply regular cycles of compression to the legs (from the ankles through to the calves and then thighs) so as to increase venous blood flow, prevent the accumulation of blood in the lower limbs and thereby decrease the risk of VTE (15). IPC has been reported to reduce the risk of DVT after gynecologic surgery when used alone or in combination with graduated compression stockings (13, 16–20). An important advantage of IPC is that it is not associated with an increased risk of bleeding. However, the majority of previous studies have focused on the use of postoperative IPC after the patient has returned to the ward, and data are lacking regarding the early use of IPC in the post-anesthesia care unit (PACU). In particular, the hemodynamic effects of IPC in the immediate peri-operative period remain unclear. Therefore, the aim of this study was to investigate the impacts of peri-operative IPC on hemodynamic indicators such as blood pressure (BP), cardiac output (CO), heart rate (HR) and systemic vascular resistance (SVR) in patients scheduled for elective laparoscopic gynecologic surgery.

## Methods

### Patients and Study Design

**Figure 1:**
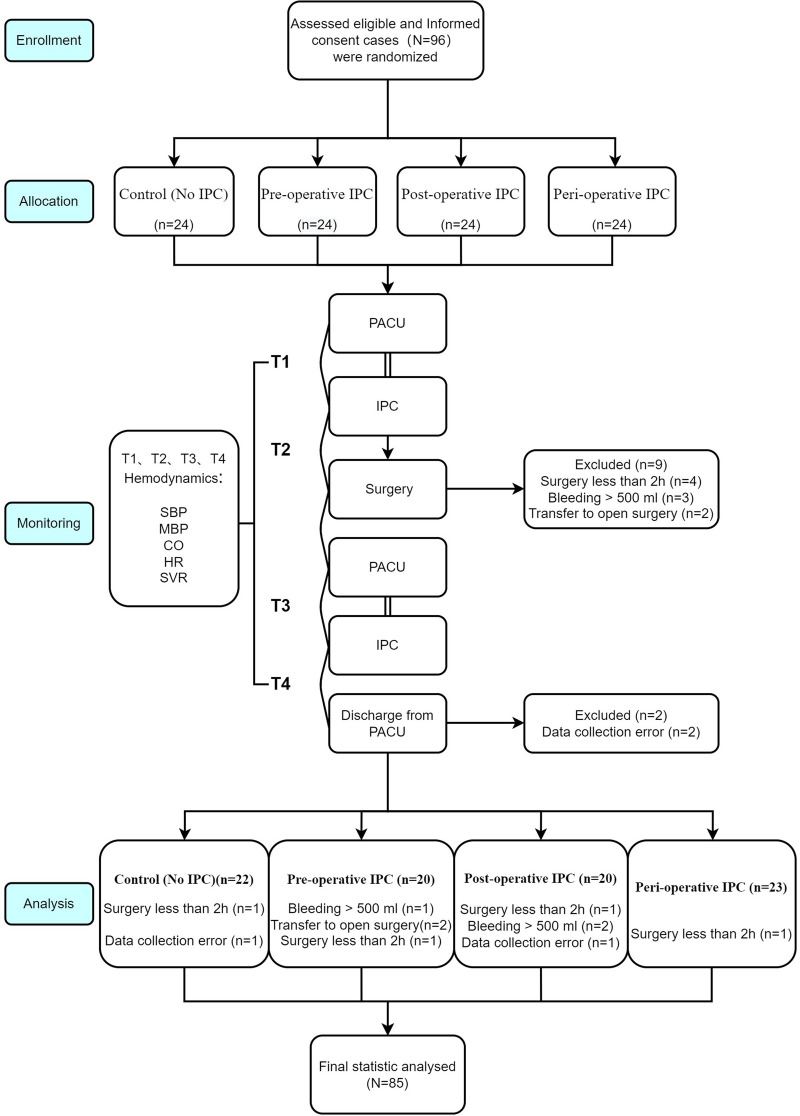
Flow chart of patients enrollment, allocation, follow up and analysis in line with CONSORT guidelines.

This randomized, controlled trial included consecutive patients scheduled to undergo laparoscopic gynecologic surgery at Shenzhen Maternity & Child Healthcare Hospital, Southern Medical University, Shenzhen, Guangdong, China between July 1, 2019 and July 31, 2021. The inclusion criteria were as follows: (1) scheduled for elective laparoscopic gynecologic surgery (such as surgery for hysteromyoma, total hysterectomy and pelvic lymphadenectomy); and (2) the expected operative time was >2 h. The exclusion criteria were: (1) hypertension; (2) hematologic disease; (3) history of thrombosis in the lower extremity veins; (4) history of surgery for varicose veins; (5) inflammation of the lower limb skin; 6) pre-operative coagulation disorder; (7) estrogen therapy; and (8) pregnancy. This study is registered at the Chinese Clinical Trial Register (ChiCTR2100044484) and was approved by the Ethics Committee of Shenzhen Maternity & Child Healthcare Hospital (SFYLS[2019]No.107). All the study participants provided informed written consent before inclusion in the trial. The study was reported in line with the 2010 CONSORT guideline for clinical trial: http://www.consort-statement.org/, and the study flow is demonstrated in Figure 1.

### Randomization and Blinding

The patients were randomized into four groups using block randomization. First, the patients were categorized into three blocks according to age (<40 years, 40–60 years and ≥60 years) with 4 patients included in each block at one time. The patients were then randomized to the following four groups using a random number table: control (IPC not used before or after surgery), pre-operative IPC (IPC performed before surgery), post-operative IPC (IPC performed in the PACU during the early post-surgical period), and peri-operative IPC (IPC performed both before surgery and in the PACU after surgery). The randomization was concealed to all statisticians involved in data analysis.

### Data Collection

The following clinical information was extracted from the electronic medical records: age, body weight, height, body mass index (BMI), operation type and operative time. Systolic BP (SBP), diastolic BP (DBP), mean BP (MBP), CO and SVR were measured at four time points: before surgery and before pre-operative IPC when given (T1); before surgery and after pre-operative IPC when given (T2); after surgery and before post-operative IPC when given (T3); just before discharge from the PACU and after post-operative IPC when given (T4).

### Interventions

All patients underwent fasting before surgery. The same general anesthesia protocol, drugs, CO_2_ pneumoperitoneum pressure and infusion volume were used for all patients. The IPC device (DSM-4S, Daesung Maref, Gunpo, South Korea) consisted of a pump and an air kit with two pairs of four-chamber balloons. This instrument can provide three different compression modes and deliver pressures from 10 to 180 mmHg. This study utilized a pressure of 60 mmHg and compression mode A, which provides sequential single-chamber inflation from the foot to the thigh followed by deflation. Each IPC treatment lasted for 30 min and was applied in the PACU immediately before and/or immediately after surgery (depending on the grouping). All IPC treatments were administered by nurse anesthetists in the PACU who had received standardized training and had more than 10 years of clinical experience. Routine IPC therapy was given on the ward after discharge from the PACU.

### Measurement of Hemodynamic Indexes

SBP, DBP, MAP, CO and SVR were measured using an automatic blood pressure monitor (CNAP Monitor 500, CN Systems, Graz, Austria). In brief, the cuff was placed on the upper arm of the patient with the arrow pointing at the brachial artery, and the finger cuffs were placed on two adjacent fingers. The required clinical information was input into the device, and the indicators were then measured.

### Endpoints and Definitions

The primary endpoint of this study was SBP before discharge from the PACU. The secondary endpoints included SBP at the other time points as well as MBP, HR, CO and SVR at T1, T2, T3 and T4.

### Sample Size Calculation

Sample size calculation was conducted in PASS software (version 15.0, NCSS, USA). A power analysis of (*α* = 0.05 and *β* = 0.85) showed that 21 patients per study group were needed to detect an effect size f = 0.4. To compensate for possible dropouts or excluded cases and satisfy the block randomization, we included 24 patients in each group.

### Statistical Analysis

SPSS 22.0 (IBM Corp, Armonk, NY, USA) was used for data analysis. The Kolmogorov-Smirnov test was applied to assess the normality of the datasets. Continuous data are described as the mean ± standard deviation if normally distributed or median (interquartile range) if non-normally distributed. Categorical data are described as frequencies and percentages. Analysis of variance (ANOVA) for repeated measurements was used to compare the primary and secondary endpoints (quantitative data) among groups and acquire *P* (time), *P* (group) and *P* (interaction). A two-sided *P*-value <0.05 was considered statistically significant.

## Results

### Clinical Characteristics of the Patients

The final analysis included 85 patients, and the baseline characteristics of the patients in the control (*n* = 22), pre-operative IPC (*n* = 20), post-operative IPC (*n* = 20) and peri-operative IPC (*n* = 23) groups are presented in Table 1. There were no significant differences between the four groups in age, weight, height or BMI (Table 1). However, operative time was significantly longer in the peri-operative IPC group than in the pre-operative IPC group (*P* < 0.05) or post-operative IPC group (*P* < 0.01). Additionally, there were significant differences among groups in operation type (*P* < 0.05).

**Table 1:** Clinical characteristics of the patients.

Characteristic	Control (*n* = 22)	Pre-operative IPC (*n* = 20)	Post-operative IPC (*n* = 20)	Peri-operative IPC (*n* = 23)	*P*
Age (years)	43.41 ± 10.60	42.15 ± 11.70	40.75 ± 10.94	41.91 ± 10.23	0.888
Weight (kg)	56.76 ± 6.20	56.09 ± 7.94	58.02 ± 7.46	55.56 ± 7.83	0.730
Height (cm)	159.91 ± 5.95	160.80 ± 5.66	160.95 ± 6.44	158.17 ± 5.58	0.388
BMI (kg/m^2^)	22.27 ± 2.75	21.65 ± 2.53	22.40 ± 2.61	22.46 ± 2.75	0.753
Operative time (hours)	3.18 ± 0.52	2.98 ± 0.72*	2.85 ± 0.69**	3.43 ± 0.79	0.035
Operation type					0.014
Myomectomy	8 (36.36%)	6 (30.0%)	6 (30.0%)	5 (21.74%)	
Hysterectomy	9 (40.91%)	6 (30.0%)	4 (20.0%)	16 (69.57%)	
Ovarian cyst	4 (18.18%)	8 (40.0%)	10 (50.0%)	2 (8.70%)	
Other	1 (4.55%)	0 (0.00%)	0 (0.00%)	0 (0.00%)	

*Data are presented as mean ± standard deviation or median (interquartile range). BMI, body mass index.*

* *P < 0.05,* **P < .01 vs. peri-operative IPC group.

### Primary Endpoint

There were no significant differences among the four groups in pre-operative SBP at T1 (Table 2). SBP exhibited no significant changes over time (T1–T4) in the control group and peri-operative IPC group (Table 2). However, SBP at T4 (just before discharge from the PACU) was significantly lower in the pre-operative IPC group (105.60 ± 3.74 vs. 113.60 ± 11.17 mmHg, *P* < 0.01) and significantly higher in the post-operative IPC group (116.65 ± 13.72 vs. 110.03 ± 12.74 mmHg, *P* < 0.01) than the corresponding pre-operative value at T1 (Table 2). Additionally, when compared with the corresponding value in the control group, SBP was significantly lower at T3 in the peri-operative IPC group (*P* < 0.05) and at T4 in the pre-operative IPC group (*P* < 0.001; Table 2).

**Table 2:** Changes in hemodynamic parameters in the four groups.

Parameter	Control (*n* = 22)	Pre-operative IPC (*n* = 20)	Post-operative IPC (*n* = 20)	Peri-operative IPC (*n* = 23)	*P*	*P* (time)	*P* (group)	*P* (interaction)
SBP (mmHg)						0.01	0.005	0.05
T1	119.00 ± 17.66	113.60 ± 11.17	110.03 ± 12.74	112.61 ± 12.59	0.194			
T2		114.95 ± 14.70		117.79 ± 17.96	0.617			
T3	118.73 ± 13.97	111.25 ± 17.47	110.85 ± 14.66	110.39 ± 13.08^#^	0.203			
T4	118.33 ± 17.17	105.60 ± 3.74**^###^	116.65 ± 13.72*	115.00 ± 10.762	0.089			
* P*	0.973	<0.001	0.005	0.115				
MBP (mmHg)						0.929	0.007	0.061
T1	83.99 ± 13.80	86.00 ± 10.80	84.92 ± 13.77	76.87 ± 12.33	0.090			
T2		83.48 ± 9.76		77.36 ± 13.86^#^	0.140			
T3	85.15 ± 13.95	84.37 ± 12.18	86.07 ± 13.69	75.65 ± 12.00^#^	0.032			
T4	83.00 ± 11.90	79.10 ± 8.53**	88.82 ± 14.17	78.61 ± 10.10	0.033			
* P*	0.574	0.048	0.378	0.401				
CO (L/min)						0.942	0.381	0.942
T1	5.33 ± 0.87	5.47 ± 0.94	5.29 ± 1.02	5.08 ± 0.90	0.410			
T2		5.41 ± 0.85		4.957 ± 0.87	0.140			
T3	5.45 ± 0.92	5.31 ± 1.18	4.98 ± 1.02*	5.22 ± 0.87	0.493			
T4	5.41 ± 0.868	5.34 ± 0.66	5.02 ± 1.07	5.27 ± 0.94	0.656			
* P*	0.688	0.733	0.071	0.460				
HR (beats/min)						0.938	0.108	0.037
T1	72.09 ± 9.33	74.85 ± 12.73	69.27 ± 11.63	68.48 ± 10.92	0.244			
T2		72.30 ± 10.10		70.14 ± 10.65	0.553			
T3	70.55 ± 11.93	70.75 ± 13.42**	67.70 ± 11.44	70.26 ± 10.98	0.834			
T4	67.53 ± 10.81*	73.70 ± 9.59^#^	64.05 ± 12.13*	73.43 ± 12.13**	0.059			
* P*	0.037	0.003	0.070	0.060				
SVR						0.326	0.156	0.631
T1	1,413.18 ± 743.79	1,213.90 ± 235.33	1,253.21 ± 351.93	1,319.48 ± 295.27	0.518			
T2		1,211.33 ± 232.96		1,362.64 ± 339.87	0.146			
T3	1,284.86 ± 339.58	1,216.60 ± 269.36	1,362.35 ± 360.83	1,454.22 ± 726.41	0.375			
T4	1,201.60 ± 325.63	1,189.20 ± 179.65	1,396.00 ± 341.07	1,298.26 ± 239.15	0.364			
* P*	0.299	0.562	0.023	0.693				

*Data are presented as mean ± standard deviation. CO, cardiac output; HR, heart rate; MBP, mean blood pressure; SBP, systolic blood pressure; SVR, systemic vascular resistance.*

* *P < 0.05,* **P < 0.01, ***P < 0.001 vs. T1; *^#^P < 0.05,* *^###^P < 0.001 vs. control group.*

### Secondary Endpoints

The other hemodynamic parameters analyzed were comparable between groups at T1 (Table 2). MBP was significantly lower at T4 than at T1 in the pre-operative IPC group (79.10 ± 8.53 vs. 86.00 ± 10.80 mmHg, *P* < 0.01) but not in the other groups (Table 2). When compared with the value at T1, heart rate was significantly lower in the pre-operative IPC group at T3 (70.75 ± 13.42 vs. 74.85 ± 12.73 beats/min, *P* < 0.01), significantly lower in the control group at T4 (67.53 ± 10.81 vs. 74.85 ± 12.73 beats/min, *P* < 0.01), significantly lower in the post-operative IPC group at T4 (64.05 ± 12.13 vs. 69.27 ± 11.63 beats/min, *P* < 0.05), and significantly higher in the peri-operative IPC group (73.43 ± 12.13 vs. 68.48 ± 10.92 beats/min, *P* < 0.001). None of the groups showed any significant differences in CO and SVR between T1 and T4 (Table 2).

## Discussion

The present study is the first to investigate whether pre-operative and/or post-operative IPC (given in the PACU) influence hemodynamic parameters in patients undergoing laparoscopic gynecologic surgery. The main finding of our research was that the use of IPC before and/or immediately after surgery resulted in only minor changes in hemodynamic parameters. Nevertheless, pre-operative use of IPC was associated with a small reduction in SBP at the time of discharge from the PACU, whereas post-operative use of IPC in the PACU was associated with a small rise in SBP. Taken together, our findings suggest that IPC in the immediate peri-operative period has no major adverse effects on hemodynamic parameters in patients undergoing laparoscopic gynecologic surgery. Additional research is needed to establish whether pre-operative use of IPC might have beneficial clinical effects such as a reduction in DVT risk.

There is a strong body of evidence to suggest that IPC can prevent DVT after gynecologic surgery whether used alone or together with graduated compression stockings (13, 16–20). However, there are limited published data regarding the effects of IPC on hemodynamic parameters during the immediate peri-operative period. To our knowledge, the present study is the first to assess whether pre-operative IPC influences hemodynamic parameters. We found that pre-operative IPC was without significant effect on SBP, MBP, HR, CO or SVR before surgery. However, pre-operative IPC was associated with lower post-operative SBP and MBP at the time of discharge from the PACU when compared with pre-operative values before the use of IPC. By contrast, post-operative use of IPC in the PACU was associated with a significant rise in SBP and a significant fall in HR. The latter findings for post-operative IPC are comparable to previously published data in healthy volunteers (21). Another study detected no significant changes in ankle SBP, DBP or MBP in healthy volunteers (22). Other published data in healthy persons and patients with congestive heart failure have suggested that IPC may increase CO without affecting heart rate due to increased preload and decreased afterload (23, 24). Our findings suggested IPC had no significant impact on CO. Overall, our findings indicate that pre-operative and/or post-operative IPC has only minor effects on hemodynamic parameters in patients undergoing laparoscopic gynecologic surgery.

Despite the minimal changes in hemodynamic parameters observed in this study, it was notable that pre-operative IPC was associated with a lower SBP at T4 when compared with the value at T1. By contrast, post-operative IPC was associated with a higher SBP at T4 (vs. T1), but this increase in SBP appeared to be prevented when IPC was also given before surgery. Nevertheless, the observed changes were small and unlikely to be clinically significant in normotensive women such as those enrolled in the present study. However, an increase in SBP after post-operative IPC would be potentially concerning in patients with high blood pressure. Hypertension is a known risk factor for DVT after gynecologic surgery (4) as well as other types of surgery (25), and the association between hypertension and DVT is thought to be mediated by vascular inflammation and endothelial cell dysfunction (26). Additionally, chronic hypotension and venous stasis predispose to DVT (27), hence any reduction in SBP following pre-operative IPC might be potentially detrimental. Therefore, further research will be needed to establish whether pre-operative and post-operative IPC are associated with changes in SBP in women with hypertension or hypotension.

The present study was not designed to investigate whether pre-operative IPC, either alone or in combination with post-operative IPC, reduced the incidence of DVT or PE in women undergoing laparoscopic gynecologic surgery. Interestingly, pre-operative IPC has been reported to decrease the incidence of DVT in patients with lung cancer undergoing video-assisted thoracoscopic lobectomy (28) and elderly patients undergoing hip fracture surgery (29). Whether or not pre-operative IPC prevents DVT in women undergoing laparoscopic gynecologic surgery will need to be investigated in a future randomized controlled trial.

Operative time was significantly longer in the peri-operative IPC group than in the pre-operative IPC or post-operative IPC groups, raising the possibility that laparoscopy duration might have been a confounding factor that affected the hemodynamic parameters. In addition to anesthesia-related changes, the elevation of intraabdominal pressure during laparoscopic surgery can lead to increases in right atrial pressure and SVR as well as a decrease in CO (30). However, the effects of laparoscopic surgery on hemodynamic parameters are rapidly reversed after surgery (30). Indeed, it was notable that the control group exhibited no significant differences in any of the hemodynamic parameters between T1 and T3. Therefore, it is unlikely that the small differences in operative time between groups confounded the analysis of the postoperative hemodynamic parameters. Nevertheless, it is well established that high-complexity procedures and longer operative time are associated with an increased risk of DVT (2–4, 7), besides, preoperative bed rest, obesity, oral contraceptives, previous episode of DVT and/or PE’ may increase the risk of DVT (31). Those patients with genetic hypercoagulopathic syndromes are also uniquely susceptible to new-onset and/or recurrent DVT and PE after surgical procedures (32), indicating that mechanical prophylaxis against DVT may be particularly important in such cases.

This study has some additional limitations. First, this was a single-center study, so the generalizability of the results remains unknown. Second, there were significant differences between groups in operative time and operation type, hence these may have been confounding factors that affected the analysis. Third, longer-term effects of IPC on hemodynamic parameters were not evaluated. Fourth, the effects of IPC on hemodynamic parameters were not compared with the effects of other types of prophylaxis such as graduated compression stockings or pharmacologic agents. Fifth, the incidence of DVT was not compared between groups to establish which protocol might be optimal for prophylaxis against VTE. Multicenter, randomized controlled trials with a longer follow-up period are needed to clarify the effectiveness and safety of pre-operative and/or early post-operative IPC in the prevention of VTE in patients undergoing gynecologic surgery.

## Conclusions

In conclusion, the administration of IPC in the PACU before surgery and/or during the early post-operative period led to only minor changes in hemodynamic parameters. However, pre-operative IPC was associated with a small reduction in SBP at the time of discharge from the PACU, whereas post-operative IPC in the PACU resulted in a small increase in SBP. Taken together, our findings indicate that IPC during the immediate peri-operative period does not have any major adverse effects on hemodynamic parameters. Therefore, additional clinical investigations are merited to evaluate whether IPC before surgery reduces the risk of DVT in women undergoing laparoscopic gynecologic surgery.

## Data Availability

The raw data supporting the conclusions of this article will be made available by the authors, without undue reservation.
